# Polyhydroxyalkanoate production from food residues

**DOI:** 10.1007/s00253-025-13554-7

**Published:** 2025-07-23

**Authors:** Simon Täuber, Sebastian L. Riedel, Stefan Junne

**Affiliations:** 1 Bioprocess Engineering, Institute of Biotechnology, Technische Universität Berlin, Ackerstraße 76, ACK 24, 13355 Berlin, Germany; 2 Environmental and Bioprocess Engineering Laboratory, Department VIII – Mechanical Engineering, Event Technology and Process Engineering, Berliner Hochschule Für Technik, Seestraße 64, 13347 Berlin, Germany; 3 Department of Chemistry and Bioscience, Aalborg University Esbjerg, Niels Bohrs Vej 8, 6700 Esbjerg, Denmark

**Keywords:** Waste animal fat, Waste cooking oil, Consumer food waste, Dark fermentation, Polyhydroxybutyrate

## Abstract

**Abstract:**

Polyhydroxyalkanoate (PHA) is an important bioplastic, its production has been commercialized, and an increase of production capacities is expected. As with many other basic chemicals, PHA production requires a currently unavailable amount of renewable carbon if bioplastic production is ever to compete with plastic production from petroleum. This extensive demand for raw materials poses challenges in terms of costs, logistics, and land use. The application of biogenic residues is therefore one of the prerequisites for any economically significant and environmentally friendly PHA production. Against this background, recent findings on the possibilities of using biogenic residues from food production and consumption to produce PHA are summarized. Waste animal fats, waste cooking oil, but also mixed food waste, either from food production or consumer food waste represent the most abundant food-related residues. They are explored for their potential to serve as substrate for PHA production. While waste animal fat and waste cooking oil can be fed directly into suspension cultures, mixed food waste can be converted into short-chain carboxylic acids from microbial hydrolysis and acidogenesis in dark fermentation before being fed. Titers and productivity of the several feedstock options are compared. The potential for economically viable and sustainable production and integration into local material cycles is highlighted, although there are still several challenges to overcome.

**Key points:**

• *Waste cooking oil enables low-cost and scalable PHA production *

• *Thermally liquefied animal fats are a suitable feed for emulsifier-free PHA production*

• *Coupling dark fermentation and PHA production is economically feasible*

• *The impact of carboxylic acid composition on PHA synthesis is explored*

## Introduction

Microbial polyhydroxyalkanoate (PHA) production has been extensively examined for its potential to replace conventional plastics with biodegradable alternatives. PHA is particularly promising in biomedical, tableware, and packaging applications (Kusuma et al. 2024), contributing to a rapidly expanding bioplastic market, which is projected to reach 98 billion USD by 2035 in comparison to 13.9 billion USD in 2024 (Future Market Insights 2025).

**Fig. 1:**
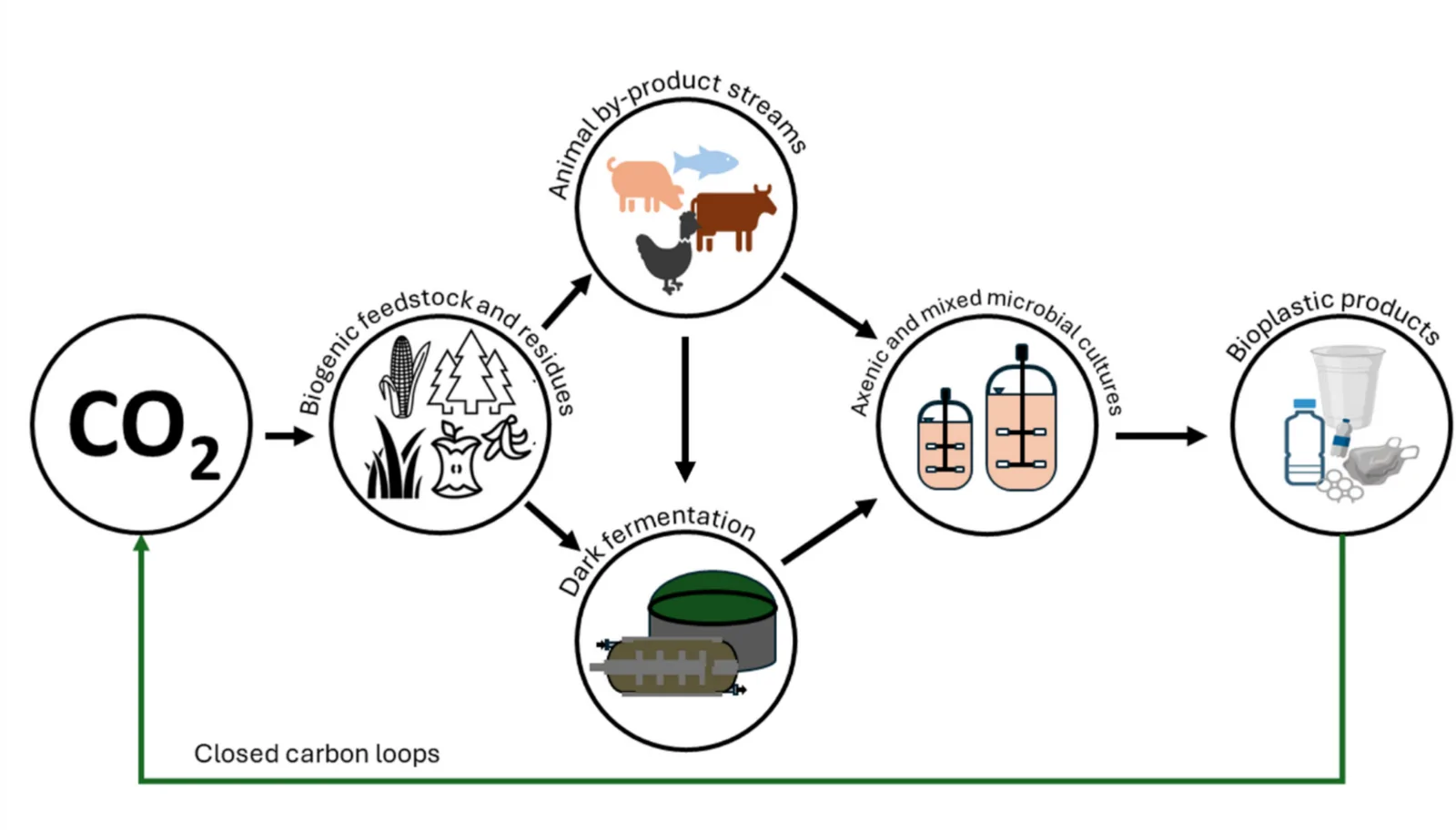
Valorization routes of food and feed residues for polyhydroxyalkanoate-based biopolymers

Large-scale PHA production is fed with renewable crops such as sugar and vegetable oils (Koller and Mukherjee 2022). Currently, production costs are still significantly higher than those of petroleum-based plastics, between 4 and 6 USD kg^–1^ compared to between 1 and 2 USD kg^−1^ (Alvarez Chavez et al. 2022; Gundlapalli and Ganesan 2025). Substrates account for up to 50% of the overall production costs (Kosseva and Rusbandi 2018), leading to intensive investigation about the use of biogenic residues as eventually cheap alternatives (Riedel and Brigham 2020; Gutschmann et al. 2022; Katagi et al. 2023). The following sections summarize recent advances in the utilization of residues from food production or consumer food waste as a contribution for low-cost carbon sources to make PHA production more affordable and sustainable (Fig. 1). The review covers the typically and widely available solid and liquid food waste fractions, that are (i) mixed solid food waste from late-stage food processing and food leftovers from consumers, (ii) waste cooking oil (WCO), and (iii) waste animal fats (WAF). Their potential to serve as carbon feed will be summarized in the following sections and critically discussed, including sustainability considerations.

### Availability of biogenic residues from food and feed production

Biogenic residues are manifold. Their market availability depends naturally on multiple factors. Many are unused or incinerated, composted, or microbially digested. It is likely that the choice of the conversion route is often not the most suitable one yet to maximize both the valorization and sustainability. Pure food processing waste fractions like WCO currently amount to between 41 and 67 Mt worldwide (Kumar et al. 2025). Based on data from the Food and Agriculture Organization of the United Nations, approximately 40 Mt of rendered animal waste, including that from pig, cattle, chicken, sheep, and goat, were produced in Europe in 2022. Of this, 5 Mt consisted of animal fats (Gutschmann et al. 2022). Using a previously published high-yield feeding strategy, up to 4 Mt of PHA could be produced annually in Europe from these waste fats (Gutschmann et al. 2023b).

Besides of WCO and WAF, unsorted consumer food waste naturally comprises a diverse composition. They can be used in anaerobic digestion (AD) to produce biogas. If the methanogenic stage is inhibited, e.g., by a pH value of 5.0 or below that, hydrolysis and acidogenesis, and eventually acetogenesis, are conducted, but no methanogenesis is present. This so-called dark fermentation (DF) is typically more robust against poor or fluctuating feedstock quality and quantity as full AD, as the hydrolytic and acidogenic bacteria are generally less sensitive against an alternating pH value or changing short-chain carboxylic acid (SCCA) concentrations than methanogenic microbes (Menzel et al. 2020). SCCAs accumulate up to an amount of about 20 gL^−1^ in DF. The acid fraction in the liquid phase often comprises acetic, butyric, and lactic acid as the most abundant SCCAs. These acids represent an interesting substrate for microbial PHA production. Hence, DF can be applied as a first stage of a process when rather mixed biogenic residues shall be made available as feedstock. The effluent of a DF or purified parts of it can then be used in a subsequent stage with an axenic culture for PHA production. Using multiple process combinations, options arise that are available for a valorization of mixed consumer food waste, a huge resource of about 132 kg per inhabitant in the EU (Eurostat 2024), a total resource of 60 Mt. As a maximum yield of 0.21 gg^−1^ SCCAs per dry mass of food waste has been reported in literature (Ashraf et al. 2023), a theoretical amount of more than 12 Mt of SCCAs could be made available for subsequent valorization in a microbial bioprocess.

### Microbial polyhydroxyalkanoate synthesis

Microbial conversion of biogenic residues for PHA accumulation follows several metabolic synthesis routes. PHAs are intracellular polyesters which are synthesized by many bacteria in response to nutrient limitation (e.g., nitrogen or phosphorus), when excess carbon is available. PHA granules serve as carbon and energy storage and support stress resistance (Obruča et al. 2022). Model organisms such as *Cupriavidus necator* (formerly known as *Ralstonia eutropha*) can accumulate PHA up to 90% of their dry cell weight (Raberg et al. 2018).

In natural isolates, PHA biosynthesis proceeds via the formation of (*R*)-3-hydroxyacyl-CoA precursors, which are polymerized by PHA synthases (PhaC). Four main metabolic routes are typically involved (Gutschmann et al. 2022):

- Sugar/amino acid pathway: Substrates like glucose or glutamate are metabolized to acetyl-CoA. The key enzymes 3-ketothiolase (PhaA) and acetoacetyl-CoA reductase (PhaB) convert acetyl-CoA into (*R*)-3-hydroxybutyryl-CoA, the precursor of PHB.
- β-oxidation pathway: Fatty acids are degraded to enoyl-CoA and further hydrated to (*R*)−3-hydroxyacyl-CoA via enoyl-CoA hydratase (PhaJ) or reduced via a β-ketoacyl-ACP reductase (FabG).
- Fatty acid biosynthesis pathway: (*R*)-3-hydroxyacyl-ACP intermediates are converted to *(R)*-3-hydroxyacyl-CoA by the *(R)*-3-hydroxyacyl-acyl carrier protein CoA-transferase (PhaG).
- Propionate pathway: Propionic acid reacts to propionyl-CoA which is, together with acetyl-CoA via a β-ketothiolase (BktB), condensed to ketovaleryl-CoA. Then, this is reduced to (*R*)-3-hydroxyvaleryl-CoA.

The polymer composition depends on the strain, the carbon source, and the specificity of PHA synthases. Most natural producers like *C. necator* form *scl*-PHA (monomers: C_3_ – C_5_), while *Pseudomonas *spp. generate *mcl*-PHAs (monomers: C_6 _– C_14_). Certain natural isolates or engineered strains can produce *scl-mcl* copolymers like P(HB-*co*-HHx) (Budde et al. 2011b; Sato et al. 2013). The carbon source has a major influence on monomer composition, and thereby on the thermal and mechanical properties of the polymer (Thiele et al. 2024). However, strain engineering increasingly enables the production of specific types of PHA independently from the feedstock, and with greater control over polymer characteristics (Chen et al. 2015; Santolin et al. 2024).

In comparison to axenic cultures, mixed microbial cultures (MMCs) tolerate complex and dynamically changing feedstock compositions even if natural isolates are applied. Therefore, they are employed in mixed waste-based PHA processes due to the comparably robust and cheap production. Processes with MMCs typically yield, however, *scl*-PHA-rich polymers (PHB, P(HB-*co*-HV)) and require enrichment strategies to stabilize the microbial community and productivity (Lorini et al. 2020). MMCs play an important role in valorizing low-value organic residues in decentralized bioproduction (Yao et al. 2025).

### PHA production from waste animal fats

WAF represents a promising carbon source for PHA production due to the high lipid content and low market value. While commonly used for biodiesel production, the high FFA content often complicates transesterification, making microbial conversion into bioplastics an attractive alternative. Table 1 summarizes recent findings on PHA production from animal-derived residues.

**Table 1:** Overview of polyhydroxyalkanoate production from waste animal fat

Microorganism	Feed	Cultivation	CDW	PHA production	Reference
*Salinivibrio* sp. M318	Waste fish oil and glycerol	Pulse-based, lab-scale bioreactor	69 gL^−1^	52% PHB^a^ 0.46 g_PHA_L^−1^ h^−1^	( Van Thuoc et al. 2019 )
*C. necator* Re2058/pCB113	Waste pork fat	Continuous feeding, lab-scale bioreactor	57 gL^−1^	80% PHA^a^	( Gutschmann et al. 2023b )
				0.63 g_PHA_L^−1^ h^−1^	
				P(HB-*co*-HHx)	
				17 mol% HHx	
*C. necator* Re2058/pCB113	Fat-/protein-emulsions	Automatic pulse-based feeding, lab-scale bioreactor	51 gL^−1^	71% PHA^a^	( Gutschmann et al. 2023a )
				0.6 g_PHA_L^−1^ h^−1^	
				P(HB-*co*3HHx)	
				20 mol% HHx	
*C. necator* Re2058/pCB113	Waste pork fat	Continuous feeding, pilot-scale bioreactor	45 gL^−1^	70% PHA^a^	( Gutschmann et al. 2023b )
				0.53 g_PHA_L^−1^ h^−1^	
				P(HB-*co*-HHx)	
				14 mol% HHx	
*C. necator* Re2058/pCB113	Waste pork/cattle fat mixture	Pulse-based, lab-scale bioreactor	45 gL^−1^	58% PHA^a^	( Riedel et al. 2015 )
				0.36 g_PHA_L^−1^ h^−1^	
				P(HB-*co*-HHx)	
				20 mol% HHx	
*C. necator* H1 G^+^3	Saturated fatty acid methyl esters from biodiesel based on hydrolyzed slaughterhouse waste	Pulse-based, lab-scale bioreactor	35 gL^−1^	80% PHA^a^	( Koller and Braunegg 2015 )
				0.94 g_PHA_L^−1^ h^−1^	
				P(HB-*co*-HV)	
				< 1 mol% HV	
*C. necator* H16	Emulsified waste pork fat	Microwell plate	14 gL^−1^	79% PHB^a^	( Riedel et al. 2023 )
*C. necator Re2058/pCB113*	Waste pork fat-protein emulsion	Shake-flask	8 gL^−1^	69% PHA^a^	( Saad et al. 2021 )
				P(HB-*co*-HHx)	
				13 mol% HHx	
*C. necator* H16	Tallow	Shake-flask	7 gL^−1^	80% PHA^a^	( Taniguchi et al. 2003 )
				P(HB-*co*-HV)	
				1 mol% HV	
*C. necator* B-10,646	Waste fish fat from smoked sprat heads	Shake-flask	5 gL^−1^	72% PHA^a^	(Zhila et al. 2023)
				P(HB-*co*-Hv-*co*-HHx)	
				2 mol% HV	
				< 1 mol% HHx	
*C. necator *B-10,646	Waste fish fat from enzymatically treated smoked spread heads	Lab-scale bioreactor	110 gL^−1^	81% PHA^a^ P(HB-*co*-Hv-*co*HHx) 2 mol% HV < 1 mol% HHx	( Kiselev et al. 2024 )
*C. necator* DSM 545 JR11	Porcine jowl	Shake-flask	4 gL^−1^	66% PHB^a^	(Rodríguez et al. 2021)
*C. necator Re2058/pCB113*	Waste pork fat-greaves	Shake-flask	4 gL^−1^	64% PHA^a^	( Saad et al. 2021 )
				P(HB-*co*-HHx)	
				13 mol% HHx	
*Pseudomonas resinovorans*	Grease trap waste	Lab-scale bioreactor	3 gL^−1^	62% *mcl*-PHA^a^	( Acedos et al. 2022 )

^a^Values rely on the proportion per cell dry weight

Any direct microbial utilization of WAF poses, however, several challenges. The high melting points (up to 60 °C) and hydrophobicity can lead to phase separation and lipid clump formation, which impair microbial accessibility in bioreactors (Riedel et al. 2015). To address this issue, lipase-secreting bacteria such as *C. necator* have been employed to hydrolyze fats and emulsify the substrate (Lu et al. 2013). Exopolysaccharide secretion has also been suggested as a mechanism to support stable emulsion formation (Gutschmann et al. 2021). In the case of small-scale early-stage process development in shake-flasks or microwell plates, emulsifying agents such as gum arabic are often added to create stable emulsions. This ensures homogeneous and reproducible starting conditions, prevents oil or fat from adhering to vessel walls or blocking gas liquid mass transfer, and improves substrate availability (Budde et al. 2011a). To avoid metabolic interference, the emulsifier must be chemically inert and compatible with microbial growth and product accumulation. However, these strategies are not easily transferable to larger scales due to increased mechanical demands for emulsification and the risk of excessive foaming or emulsion breakdown during high-cell-density (HCD) fermentations (Riedel et al. 2014). A recently proposed standardized workflow enables small-scale screening of WAF-based PHA processes, considering well-plate and shake-flask design, oxygen transfer, and mechanical pre-emulsification (Riedel et al. 2023). A particular emphasis was put on avoiding fat deposits on walls, which reduces the bioavailability of feedstock, a rather underexplored challenge in early process development with fat substrate. Additionally, the liquid flow field changes when fat is emulsified and assimilated while the cell concentration changes. It is therefore not clear whether the common knowledge about the applicability of flask and well design is transferable to cultures with fat substrate.

For scale-up, a robust, emulsifier-free feeding strategy was introduced that uses thermally liquefied pork fat. The fat was continuously dosed into lab- and pilot-scale reactors (Gutschmann et al. 2023b). Starting from a low concentration of emulsifiable oil (10 gL⁻^1^), *C. necator* was able to establish stable growth and emulsification, leading to > 45 gL⁻^1^ PHA, with space–time yields of 0.63 gL⁻^1^ h⁻^1^ and polymer contents above 70%. Beyond WAF, bone-based residues (~ 5% residual fat) have also been evaluated. Though PHA polymers with high 3-hydroxyhexanoic acid (HHx) content (> 20 mol%) were produced, the solids required for sufficient biomass loading complicate downstream processing and are therefore of limited scale-up potential (Saad et al. 2021). In addition, the intrinsic nitrogen content of such residues must be carefully considered when nitrogen limitation is employed as the metabolic trigger for polymer synthesis.

To support process development and optimization, a kinetic model for P(HB-*co*-HHx) synthesis from WAF was introduced (Ochoa et al. 2025). It describes cultures with an engineered *C. necator* strain with the ability to synthesize the HHx copolymer from oleaginous sources. The model, which was validated in high cell density fed-batch fermentations (> 100 gL⁻^1^ CDW), accurately predicts biomass and polymer production and supports control strategies by critical parameter identification.

### PHA production from waste cooking oil

WCO is another lipid-rich residue considered as suitable for PHA production. It is readily available after being used in food preparation, but its use is in competition with other, already established production chains, including biodiesel production, animal feed (after appropriate treatment), and in industrial oleochemistry, where it serves as a raw material for the manufacture of lubricants, soaps, and surfactants (De Feo et al. 2023). These competing applications can significantly affect both, the availability and price of WCO. Nonetheless, low-grade WCO with impurities has proven suitable for microbial conversion into PHA, especially in *C. necator* cultivations. Such oils have been shown to support high PHB accumulation and can be an effective, and even affordable substrate for bioplastic production (Jiang et al. 2025).

An overview of relevant studies using both, pure and mixed cultures for WCO-based PHA synthesis, is provided in Table 2. In comparison to solid fats, WCO is easier to dose and emulsify, but may exhibit also high batch variability due to its heterogeneous origin and composition in affordable low-quality fractions. Like other oleaginous substrates, it requires, however, enzymatic hydrolysis and emulsification to become bioavailable. Fortunately, the mixing intensity in typical operation of aerobic bioprocesses creates a surface area which is sufficiently large for enzymatic degradation, especially in stirred-tank bioreactors.

**Table 2:** Overview of polyhydroxyalkanoate production from waste oil

Microorganism	Feed	Cultivation	CDW	PHA production	Reference
*Bacillus thermoamylovorans* PHA005	4 vol% WCO	Batch Shake-flask cultures	4 gL^−1^	87.5% PHA^a^ P(HB-*co*-HV) 6.4 g_PHA_L^−1^ h^−1^	( Sangkharak et al. 2021 )
*C. necator* Re2058/pHT_1-CBP−M−CPF4_	1 vol% mixed WCO fractions	Batch Shake-flask cultures	8.5 gL^−1^	75% PHA^a^	( Fook et al. 2024 )
				P(HB-*co*-HHx)	
				14 mol% HHx	
				114 g_PHA_L^−1^ h^−1^	
*C. necator* H16	WCO and waste fish oil	Fed-batch Lab-scale bioreactor	135 gL^−1^	77% PHA^a^ 1.73 g_PHA_L^−1^ h^−1^	( Loan et al. 2022 )
*C. necator* H16 Δ(A3043/lipAB)	WCO (crude)	Fed-batch 200-L pilot-scale	> 200 gL^−1^	194 gL^−1^ PHA 4.05 g_PHA_L^−1^ h^−1^	( Jiang et al. 2025 )
*Pseudomonas chlororaphis* 555	WCO	Batch Lab-scale bioreactor	73 gL^−1^	26% PHA^a^ 13.9 g_mclPHA_L^−1^ 0.29 g_mclPHA_L^−1^ h^−1^ 18.324 Da	( Ruiz et al. 2019 )
*Pseudomonas alcaligenes*	Food waste oil	Fed-batch Lab-scale bioreactor	16 gL^−1^	54% PHA^a^ 0.23 g_PHA_L^−1^ h^−1^ 54.782 Da	( Pan et al. 2021 )
MMC	WCO	Sequencing batch Lab-scale bioreactor	1.8 gL^−1^	38% PHA^a^ *scl-* and *mcl-*PHA < 8% HHx	( Tamang and Nogueira 2021 )

^a^Values rely on the proportion per cell dry weight

Jiang and colleagues demonstrated scalable PHA production from untreated WCO in a 200 L stirred-tank reactor. The fermentation achieved PHA titers exceeding 194 gL⁻^1^ within 48 h and a carbon conversion efficiency above 86%. The process was implemented without extensive feedstock pretreatment, relying instead on moderate lipase enhancement and turbulent mixing. The same strain was used in a 150-m^3^ industrial fed-batch process with food-grade palm oil, reaching a PHA titer of 264 gL⁻^1^ (Jiang et al. 2025). These findings underline the general scalability, a pre-requisite for any fast industrial implementation.

### PHA from dark fermentation effluent

If waste fats and oils cannot be directly used, for example, when they are part of mixed food waste fractions, or mixed late stage food processing waste and consumer household waste, they can be made available by prior microbial conversion through hydrolysis and acidogenesis in DF. Aside of a separate DF, the same process can be conducted as a first stage in a typical full anaerobic digestion as applied sometimes in biogas production (Menzel et al. 2020; Janesch et al. 2021). The resulting dark fermentation effluent (DFE), which is rich in SCCAs, may represent a valuable substrate for PHA production in both, MMCs and axenic suspension cultures, thereby expanding the spectrum of applicable food waste fractions. Several recent studies described the utilization of DFE for PHA production, utilizing subsequently MMCs or axenic cultures at laboratory and pilot scale. An overview of typical process performance parameters as provided in recent publications is given in Table 3.

**Table 3:** Overview of polyhydroxyalkanoate production from dark fermentation effluent as feed

Microorganism	Feed	Cultivation	CDW	PHA production	Reference
*C. necator* DSM 545	Apple pomace and potato peel: HBu 4.2 gL^−1^ HAc 3.0 gL^−1^ HPr 0.8 gL^−1^ HCa 0.65 gL^−1^ VFA 8.8 gL^−1^	Semi-continuous 2.5 L immersed membrane bioreactor	3.3 gL^−1^	54.5% PHA^a^ P(HB-*co*-HV)	( Vu et al. 2023 )
*P. putida KT2440*	Cafeteria food waste: 35.9 g_COD_L^−1^ HAc 5.1 gL^−1^ HBr 4.8 gL^−1^ HPr 2.4 gL^−1^	Batch 100 mL continuously mixed bottles		56% PHA^a^ 60 mol% HB < 10 mol% HV	( Chandra et al. 2023 )
MMC	Food waste and sewage sludge (30:70)	Fed-batch Lab-scale bioreactor	10.6 gL^−1^	31.4% PHA^a^ 3.3 g_PHA_L^−1^ 97 mol% HB, 3 mol% HV	(Perez-Zabaleta et al. 2021)
MMC	Vegetable waste: SCCA 13.4 gL^−1^ HAc 7.3 gL^−1^ HBu 5.3 gL^−1^ HVa 0.8 gL^−1^	Sequencing-batch Lab-scale bioreactor	2.82 gL^−1^	28% PHB^a^ 0.78 g_PHB_L^−1^	( Kumar et al. 2021 )
MMC	Dairy waste: SCCA 203 mmolL^−1^ HAc 54.7% HBu 36.3% HLa 6.5%	Fed-batch Lab-scale bioreactor	-	62% PHB^a^	(Colombo et al. 2019)
MMC	Dairy waste: SCCA 14.6 gL^−1^ HAc 8.0 gL^−1^ HBu 5.3 gL^−1^ HLa 0.95 gL^−1^	Fed-batch DO controlled, 1 L bioreactor	-	35% PHA^a^ P(HB-*co*-HV) 34 mol% HV 0.2 gL^−1^ h^−1^	( Asunis et al. 2022 )
MMC	Cheese whey: HAc 4.0 gL^−1^ HBu 1.0 gL^−1^ HPr 0.65 gL^−1^ HVa 0.21 gL^−1^	Fed-batch DO controlled, 2 L bioreactor	-	45% PHA^a^ P(HB-*co*-HV) 24 mol% HV	( Lagoa-Costa et al. 2022 )
MMC	Fruit juice: HBu 8.9 gL^−1^ HLa 2.3 gL^−1^ HAc 0.6 gL^−1^ HCa 0.5 gL^−1^	Batch Shake-flask culture	-	43.3% PHA^a^	( Kora et al. 2023 )
MMC	Fruit waste: HCa 4.6 gL^−1^ HBu 0.96 gL^−1^ HAc 0.90 gL^−1^	Fed-batch DO controlled, 60 L bioreactor	-	71.3% PHA^a^ HB/HV/HHX 33/1/66 mol% 3.3 g_COD_L^−1^ h^−1^	( Silva et al. 2022 )
MMC	Cheese whey: 18.7 g_COD_L^−1^ h^−1^	Fed-batch DO controlled, 6 L bioreactor	-	50% PHA^a^ 0.33 g_PHA_L^−1^ h^−1^ P(HB-*co*-HV) 27 mol% HV	( Carvalheira et al. 2022 )
MMC	OFSMW (30%)/sewage sludge (70%): 36 g_COD_L^−1^	Fed-batch DO controlled, 100 L bioreactor	-	3.2 g_COD-PHA_L^−1^ 51% PHB^a^	( Moretto et al. 2020 )
MMC	OFSMW (30%)/sewage sludge (70%): 20 g_COD_L^−1^	Fed-batch 80 L bioreactor	-	39% PHA^a^ 0.075 g_PHA_L^−1^ h^−1^	( Valentino et al. 2020 )
MMC	Fruit waste: HBu 1.4 gL^−1^ HAc 1.1 gL^−1^ HPr 0.25 gL^−1^ Total COD 5.4 gL^−1^	Fed-batch 60 L bioreactor	2.1 gL^−1^	80.5% PHA^a^ 0.34 g_PHA_L^−1^d^−1^ P(HB-*co*-HV) 24 mol% HV	( Matos et al. 2021 )
MMC	OFMSW: 14.6 g_COD_L^−1^ HAc 4.5 gL^−1^ HBu 2.1 gL^−1^ HPr 1.3 gL^−1^ HVa 1.2 gL^−1^	Sequencing-batch 3 L bubble column reactor	52 gL^−1^	16.9% PHA^a^ 18.4 g_PHA_L^−1^ 0.04 g_PHA_L^−1^ h^−1^	(Rojas-Zamora et al. 2023)

^a^Values rely on the proportion per cell dry weight

The composition of SCCAs in DFE plays a central role in determining the monomer portion and material properties of the resulting PHA. Even-chain acids like acetic acid (Hac) and butyric acid (HBu) promote the formation of 3-hydroxybutyric acid (HB) monomers. This leads to polymers that are highly crystalline, but brittle. In contrast, odd-chain acids such as propionic acid (HPr) and valeric acid (HVa) lead to incorporation of 3-hydroxyvaleric acid (HV) monomers, which increase flexibility, reduce brittleness, and improve processing characteristics (Silva et al. 2022; Mai et al. 2024). The link between substrate and polymer structures enables the tuning of material properties by adjusting the SCCA profile in DFE through the choice of substrate or distinct fermentation conditions (Menzel et al. 2020). As such, feedstock composition directly influences both, microbial performance and product functionality (Brigham and Riedel 2018). Several reviews about PHA monomers and their characteristics have been published (Miyahara et al. 2021; Park et al. 2024; Mai et al. 2024).

Recent studies showed that supplementing DFE with HVa in concentrations between 11 and 63% resulted in a proportional incorporation of HV into the polymer, demonstrating a linear correlation between precursor concentration and monomer composition. Notably, this modification did not affect process performance, as PHA productivity remained constant at 0.33 g_PHA_L⁻^1^ h⁻^1^, while the yield reached consistently 0.75 g_COD-PHA_ g_COD-SCCA_^−1^ (Carvalheira et al. 2022). Comparably high costs of HVa among suitable SCCAs limit, however, any economically viable application. While HAc prices are between 300 and 450 USD t^−1^ (Gong et al. 2024), HVa prices can exceed 2500 USD t^−1^, depending on the compound’s purity and production site (Kim et al. 2019). When caproic acid (HCa) served as the main carbon source, a comparable shift of the monomer composition was observed for the polymers’ HHx content (Silva et al. 2022). In another study, two varieties of fermented cheese whey, the liquid byproduct from milk coagulation and fermentation during cheese production, together with a different SCCA composition were applied as carbon source for PHA production (Colombo et al. 2019). While one fraction contained HLa, HAc, and HBu (58%, 16%, and 26%), the other was composed of HAc, HPr, HBu, HLa, and HVa (58%, 19%, 13%, 6%, and 4%). The findings indicated that the first fraction was suitable in producing PHB, whereas the second led to the production of PHA that contained 40 mol% HV- and 60 mol% HB-monomers. The dependence between SCCA feed composition and product feature enables a targeted polymer production in case the composition and concentration of SCCAs are controllable. However, achieving a consistent SCCA profile in DFE remains an ongoing research challenge, as it depends on multiple factors such as substrate variability and fermentation conditions. Any optimal operation state can vary in dependence on the most abundant and active microbial consortia members.

MMCs can be adapted to utilize DFE by applying selection strategies that favor organisms capable of converting the most abundant SCCAs in the DFE into PHA. One widely used method is the feast and famine strategy. In this approach, MMCs are exposed to alternating phases of carbon availability: During the feast phase, the culture is supplied with an excess of carbon, like glucose, HAc, or directly with DFE. All these substrates are usually assimilated rapidly while PHA accumulates. This phase is followed by a famine phase, where the external carbon source depletes. During this stage, cells that have previously stored PHA gain a growth advantage. Applying alternating cycles, a selection to achieve a storage capacity of up to 80% PHA was achieved (Oliveira et al. 2017; Silva et al. 2022). A repeated batch cultivation mode with MMCs can lead to PHA-rich biomass harvest after a prior settling phase (Kumar et al. 2021; Muhorakeye et al. 2022; Rojas-Zamora et al. 2023). Since a part of the previously accumulated PHA is consumed during fasting, it is beneficial to separate PHA production. Then, the MMC is firstly produced and enriched using the feast and famine strategy and subsequently transferred as inoculum to a second fed-batch reactor for tailored PHA production while both stages are fed with DFE (Valentino et al. 2017).

Such process workflows have been tested at pilot scale with various DFEs marking an important step towards industrial biorefinery applications. The following section will focus on the most recent studies in this area under consideration of DFE and PHA composition, titer, yield, and productivity. It was demonstrated, for example, that sheep cheese whey is an effective substrate for PHA production within a three-stage process, despite its high protein content leading to a low C/N ratio. The effluent proved suitable for enriching the MMC without the need for additional nutrients, achieving a PHA content of 35% with a polymer composition of 66/34 for HB/HV and a productivity of 0.2 gL^−1^ h^−1^. Notably, the DFE was employed without further pre-treatment following a settling phase to remove the solid fraction (Asunis et al. 2022). A similar pilot-scale process design was used to produce PHA with varying HV content, applying untreated effluent from fermented cheese whey and adding HVa as HV precursor for tailored PHA composition. While pure cheese whey resulted in a polymer containing 28% HV and an overall cellular PHA concentration of 50%, the introduction of HVa increased the HV fraction to 62%. An average productivity of 0.33 g_PHA_L^−1^ h^−1^ was reached (Carvalheira et al. 2022). A three-stage process was optimized for producing polymers rich in HHx, utilizing caproate-rich effluent at pilot scale (Silva et al. 2022). Fruit juice was fermented in a 100 L up-flow anaerobic sludge blanket reactor, producing effluent with a concentration of 12.9 g_COD_L^−1^ SCCAs. HCa constituted 73.5% of the total acids. Following an enrichment stage for a MMC, filtered DFE was subsequently fed with a DO-controlled pulse feeding strategy for PHA production. A PHA content of 71.3% and a composition of HB/HV/HHx % of 33/1/66 were achieved with a production rate of 3.29 g_COD_L^−1^ h^−1^, representing one of the highest rates among those reported for polymer production, rich in HHx, from DFE at pilot scale. Matos and colleagues applied effluent which first was decanted to remove solids from fermented fruit waste in a three-stage process in a pH-auxostat fed-batch mode. A maximal PHA content of 80.5% was achieved, following the effluent feeding with a composition of 21.3% HAc, 58% HBu, 6.7% HVa, and 8.7% HPr, with a productivity of 0.34 gL^−1^ h^−1^ (Matos et al. 2021).

A pilot plant was established applying sludge from a wastewater treatment plant (WAS) and the organic fraction of municipal solid waste (OFMSW), mainly composed of biodegradable household and food waste mixed in a 70/30 ratio (Moretto et al. 2020). The resulting SCCA concentration was 3.5 g_COD_L^−1^. As part of the process, the effluent was centrifuged and filtered prior to utilization in a MMC. In this process, an intracellular PHA content of 51% was achieved. Further calculations showed that 259 L of OFMSW-WAS mixture, corresponding to 7.8 kg produced COD_SCCA_, are required to produce 1 kg of PHA (1.7 kg of COD_PHA_) at a productivity of 0.4 g_COD-PHA_L^−1^ h^−1^. An economic evaluation estimated the annual PHA production at 81 t, with a revenue increase of 23% compared to traditional co-digestion for biogas production. The same setup was examined under the consideration of fluctuating temperatures, concluding that stability and production capacities of the MMC can be maintained by adjusting the feeding rate (Valentino et al. 2020). It was further demonstrated that PHA production can be integrated with hydrogen and succinic acid production at laboratory scale (Amulya and Venkata Mohan 2022). Utilizing effluent from synthetic wastewater, PHA was produced with a final content of 23%. The CO_2_ that was emitted during DF was directed into another bioreactor to enhance succinic acid production by 16%. This approach illustrates the potential for a comprehensive carbon cycle concept and further potential for process coupling in any biorefinery concept.

While MMCs pose a challenge for process control and reproducibility, axenic cultures are typically applied when a defined and consistent PHA composition is needed. This enables more predictable and reproducible processes, which are particularly important for industrial applications. Such cultures can include both genetically modified organisms and natural isolates. However, studies that describe the investigation of DFE as a substrate in axenic cultures remain scarce. Several key challenges need to be solved when applying DFE as feed for PHA production. One major issue is the low carbon concentration, which leads to dilution of the cell suspension in a fed-batch mode. This can be addressed by applying cell retention systems or the separation of SCCAs from DFE prior to feeding. The latter method, however, would also eventually separate SCCAs from other nutrients, which will be lost for the PHA production.

It was demonstrated that *C. necator* can utilize SCCAs from DFE (Domingos et al. 2018; Vu et al. 2022). To circumvent the low carbon content of DFE - in comparison to conventional carbon feed - and thus a dilution of biomass and product, Vu and colleagues employed a 2.5 L immersed membrane bioreactor for PHA production (Vu et al. 2023). The effluent, which was derived from apple pomace and potato peel fermentation, achieved a total SCCA concentration of 8.81 gL^−1^. *C. necator* was cultivated with pulse-wise feeding and medium withdrawal. However, due to the low SCCA concentration, no growth was achieved after 80 h of cultivation, and PHA was utilized rather than accumulated. Nonetheless, the application of cell retention systems may be a key to enhance the overall process performance, as described by other authors for coupling DFE and other value-added bioprocesses (Chalima et al. 2021).

Another problem is the low pH value of typical DFE, which can inhibit microbial activity during PHA production. This can be mitigated by employing a pH-auxostat strategy to dynamically control and stabilize the pH during cultivation and avoid an inhibiting SCCA concentration. Since DFE originates from mixed microbial cultures, sterility cannot be ensured, posing a risk of contamination in axenic cultures. It is then important to keep a certain growth advantage, e.g., high tolerance against nitrogen or phosphate limiting conditions during the phase of DFE feeding. Lastly, DFE often contains a high number of suspended solids, which must be removed to prevent any interference with downstream applications. This is typically conducted via centrifugation, sedimentation (Kong et al. 2020) or filtration. Scalability of retention systems follows membrane bioreactor developments, which are in a typical industrial application range of several hundred m^3^ of cultivation volume (Xiao et al. 2019). Other challenges as typical for large-scale microbial bioprocesses remain such as liquid phase heterogeneity. This is generally a subject of intensive research in upstream bioprocess development (Neubauer and Junne 2016).

### Cost analysis

Substrate costs are naturally contributing a lot to the total bioproduction costs. In case of food waste, however, no generally valid cost estimation can be conducted. Depending on the local demand and supply chains, food waste might be available at low or no costs in comparison to substrate from renewable crops. Any clear statement is, however, almost impossible for mixed consumer food waste due to the lack of stock market prices. Nevertheless, several stock market prices are available in case of WCO: they reached to about 1.25 USD kg^−1^ in early 2025. This is rather high, while even crude palm oil has lower stock market prices of about 0.85 USD kg^−1^ (Argus 2025). Yields vary across oil fractions and microorganisms. Under consideration of a yield of between 0.25 and 0.8 gg^−1^ PHA per oil-based substrate (Lim et al. 2023), substrate costs of between 3.4 and 1.0 USD kg^−1^ of PHA can be allocated.

Prices of WAF depend on the fraction and quality; they are often agreed individually in local markets. Category 3 (low-risk waste material that was originally intended for human consumption) pork lard prices are in the range of 1.1 USD kg^−1^ in Northwestern Europe, prices for mixed fat are about 10% lower (Watts 2025). Substantially lower prices can be assumed for non-edible fat fractions. Assuming a market price of 0.2 USD kg^−1^ (Riedel et al. 2015) and a yield of 0.33 gg^−1^ PHA per WAF (Table 1), the substrate costs will amount for a range of between 0.6 and 3.3 USD kg^−1^ of PHA.

Costs of mixed food waste can be assumed as being neutral (companies are paid for waste collection) or up to about 100 USD t^−1^ according to average costs for waste collection in the EU (Nohales and Stinavage 2024). Equivalent prices of DFE are difficult to guess. The stock market prices of pure HAc are up to about 0.75 USD kg^−1^ as of 2025 in Central Europe, prices for technical grade HAc have been reported as being between 0.3 and 0.45 USD kg^−1^ (Gong et al. 2024). At a yield of 0.02 gg^−1^ PHA per HAc (Kacanski et al. 2023), maximum substrate costs are 37.5 USD kg^−1^ of PHA. A comparison of PHA production and full AD, in which HAc can be added for methane production, does not attract for the application in material use at a first glance: the yield of methane from HAc is about tenfold greater (Lemaigre et al. 2023). If a reimbursement of 3 USD kg^-1^ of methane is applied based on current revenues as in Germany in 2025, AD would currently be economically advantageous. In the case of DFE, a yield of 0.03 gg^−1^ PHA per DFE is assumed. If approximately 5% of the investment costs of a complete AD plant were spent on the hydrolysis/acid formation in a separate DF stage (own cost assessments), the costs for providing DFE (substrate costs) amount to approximately 1.8 USD kg^−1^ PHA. As mentioned before, PHA production costs beyond substrates account for about 1 to 3 USD kg^−1^, that is half of the total production costs. Techno-economic analyses revealed that production costs vary between 1.4 and 1.6 USD kg^−1^ (Shahzad et al. 2017). Then, a selling price similar to that of PHA from palm oil between 2 and 4 USD kg^−1^ (Gundlapalli and Ganesan 2025) is achievable, which leads to the conclusion of a general economic feasibility of DFE as substrate. All the calculations imply, however, a similar process and product quality as achievable with palm oil.

While AD is a competing and eventually more beneficial process under certain market circumstances in some regions of the world, a share between material and energy use may have practical benefits in biorefineries and offer alternatives in case of dynamic markets. It will be important, however, to reduce separation efforts and costs as much as possible while taking the advantage of manifold substrate components in the DFE. This will make it more valuable as feed for a subsequent bioprocess than purified acids, and keep the consumption of energy and water, and finally overall operational expenditures low.

### Sustainability assessments

Naturally, any sustainability measures of any biotechnological process depend highly on the regional feedstock production and supply chain. The system boundaries include the whole agricultural production and even seasonal weather conditions if primary renewable resources like sugars are used as feed. A direct comparison of studies may not be valid for a process conducted with a similar feedstock and process performance, but under otherwise different conditions like the production site’s location, and supply chain of food waste fractions, including the collection and transport. The rare life cycle assessment (LCA) studies that describe the application of food waste or similar waste fractions for PHA production should, however, not remain unmentioned. A LCA with regrind pasta, an industrial food production waste fraction, yielded a manifold impact of the downstream processing on climate relevant emissions per PHA mass than the upstream part. The upstream part included SCCA filtration from DFE via membrane filtration (Saavedra del Oso et al. 2023). If compared to AD, PHA has a worse environmental performance, as an assessment yields 44.8 and 35.7 kg_CO2-eq_t^−1^ of cheese whey either used for PHA production or full AD (Asunis et al. 2021). The same report states the importance of the energy sources on the assessment’s outcome, while the direct impact of altered energy sources was compared elsewhere (Vogli et al. 2020). The authors concluded that a partial use of energy from bioproducts, here pyrolysis, had already a big effect on reducing the overall environmental impact of the bioproduction. Other studies confirm that PHA production from slaughtering waste does have a 12% lower environmental impact with respect to relevant greenhouse gas emissions than the production of conventional low-density polyethylene if renewable wind power was applied (Ali et al. 2023). These attempts demonstrate that an increasing share of renewable, low-emission energy sources together with suitable concepts of energy sharing *on site* has the potential to compensate a lot product-specific emission of PHA production.

## Conclusion

It has been shown that animal by-product streams such as WAF, non-liquefiable fat/protein-emulsions, fat separator waste, WCO, and mixed food waste from late-stage food processing and consumer food waste, are suitable resources for the application as feedstock for PHA production. While waste residues carry a comparably lower environmental burden as renewable crop sources and do not compete with food and feed production, the logistic network and infrastructure to collect it for bioproduction are underdeveloped. Availability and costs are often unclear, supply chains are not established, which altogether hinders investments. Additionally, while a decentralized conversion of waste might be possible already to some extent in the present infrastructure, e.g., for full AD, decentralized production of bioplastics might not be economically and technically viable within a short term. It requires a new production model which is untypical for the chemical industry so far and might not have a similar support by stakeholders as traditional centralized production. Process coupling for parallel production of several products remains as an additional option to increase revenues (Yadav et al. 2021).

Another challenge remains on the level of microbial synthesis with varying quality and quantity of residual resources. This can only be resolved if processes are established in which biogenic residues of different qualities become well manageable to achieve PHA with almost similar attributes. Little has been published so far about monitoring intracellular PHA synthesis in real-time, although a pre-requisite to increase process robustness. Among the rare examples is the application of photon density wave spectroscopy to quantify intracellular PHA enrichment in suspension culture (Gutschmann et al. 2019). The integration of suitable process analytical technologies becomes even more important in decentralized production concepts, often assisted with remote control, or in continuous processes, which are suited for a low concentrated feed like DFE.

Finally, a system-based policy framework that considers all options to resolve the current substrate supply issue of commodity biochemicals is required to ensure any practical implementation of the many findings summarized in this review (Gundlapalli and Ganesan 2025).

## Data Availability

Not applicable.

## Abbreviations

AD: Anaerobic digestion
COD: Chemical oxygen demand
DF: Dark fermentation
DFE: Dark fermentation effluent
DO: Dissolved oxygen
FFA: Free fatty acid
HAc: Acetic acid
HB: 3-Hydroxybutyric acid
HBu: Butyric acid
HCa: Caproic acid
HHx: 3-Hydroxyhexanoic acid
HLa: Lactic acid
HPr: Propionic acid
HV: 3-Hydroxyvaleric acid
HVa: Valeric acid
*Mcl*-PHA: Medium-chain-length-polyhydroxyalcanoate
MMCs: Mixed microbial cultures
OFMSW: Organic fraction of municipal solid waste
PHA: Polyhydroxyalkanoate
PHB: Poly-3-hydroxybutyrate
P(HB-*co*-HV): Poly(3-hydroxybutyrate-*co*-3-hydroxyvalerate)
P(HB-*co*-HHx): Poly(3-hydroxybutyrate-*co*-3-hydroxyhexanoate)
SCCAs: Short-chain carboxylic acids
*Scl*-PHA: Short-chain-length-polyhydroxyalcanoate
WAF: Waste animal fats
WAS: Waste activated sludge
WCO: Waste cooking oil
